# Parental Socioeconomic, Demographic, and Neonatal Factors Influencing Infants' Low Birth Weight: A Cross‐Sectional Study in Bangladesh

**DOI:** 10.1002/hsr2.71146

**Published:** 2025-08-27

**Authors:** Md. Rasel Hossain, Susmita Begum, Mahmuda al Neyma, Kabir Hossain, Mohammad Omar Faruk, Sorif Hossain, Humayra Afnan

**Affiliations:** ^1^ Department of Statistics Noakhali Science and Technology University Noakhali Bangladesh; ^2^ Institute for Intelligent Systems Research adn Innovation Deakin University Waurn Ponds Australia

**Keywords:** Bangladesh, demographic, low birth weight, preterm birth, socioeconomic

## Abstract

**Background and Aims:**

Low birth weight (LBW) is a critical indicator of infant survival and a reflection of significant public health challenges. This study aims to investigate the parental socioeconomic, demographic, and neonatal factors influencing LBW among newborns in Bangladesh.

**Methods:**

Simple random sampling was used to gather data for the study, which included 597 recent births. The *χ* ^2^ test was used to evaluate associations. Binary multivariate logistic regression was used to identify the risk factors for LBW, with a significance level set at a *p* value of 0.05.

**Results:**

The results indicated that 27.5% (*n* = 164) of the infants had LBW. The binary logistic regression analysis showed, preterm birth was associated to a twofold increase in the odds of LBW (OR: 2.376, 95% CI: 1.566–3.606, *p* < 0.05). Furthermore, maternal age had a significant impact on birth weight; mothers aged > 35 years had a 39.6% lower chance of having a LBW child than mothers < 21 (OR: 0.396, 95% CI: 0.252–0.624, *p* < 0.05). The odds of giving birth to a low‐weight baby were 39.1% lower for women who took certain medications during pregnancy than for those who did not (OR: 0.609, 95% CI: 0.373–0.995, *p* < 0.05). LBW is 1.838 times more likely to occur in babies born to mothers who have mineral deficiencies (OR: 1.838, 95% CI: 1.200–2.816, *p* < 0.05).

**Conclusion:**

The risk of LBW is greatly increased by premature birth, younger maternal age, mineral deficiencies, and the absence of maternal medication use during pregnancy. Reducing the prevalence of LBW and enhancing neonatal health outcomes in Bangladesh requires addressing these factors.

## 1 Introduction

Low birth weight (LBW) is a serious worldwide health issue that has an impact on the health of infants and their mothers. The World Health Organization (WHO) defines LBW as a birth weight of less than 2500 g (5.5 pounds), regardless of gestational age, that resulted from preterm birth (before 37 weeks of gestation), intrauterine growth restriction, or a combination of both [1]. According to estimates, the prevalence of LBW is 14.6% globally, with low‐ and middle‐income nations accounting for 91% of LBW cases, mostly in Southern Asia (48%) and sub‐Saharan Africa (24%) [2]. LBW is responsible for 60%–80% of neonatal morbidity and mortality, making it a crucial indicator of neonatal health. The World Health Organization reported that neonatal with LBW are noticeably more likely to die than those with average birth weight. LBW babies are more likely to experience birth asphyxia, stunting, malnourishment, infections, hypothermia, hypoglycemia, and long‐term disabilities like learning disabilities, seizures, and cerebral palsy [3, 4, 5, 6, 7]. Additionally, LBW is closely linked to the later onset of non‐communicable diseases like diabetes, high blood pressure, and cardiovascular disorders [3, 6].

Low birth weight (LBW) affects more than 20 million newborns globally each year, making up around 15%–20% of all births [8]. A significant proportion (95.6%) of low‐weight births worldwide occur in developing nations [9]. The prevalence of LBW is only 7% in developed regions, but it is 16.5% in developing countries [9]. The highest prevalence is found in South‐Central Asia (27%), followed by sub‐Saharan Africa (15%) and other regions (10%–14%), including Central and South America and Oceania [9]. According to the 2017–2018 Bangladesh Demographic and Health Survey (DHS), 16% of newborns in Bangladesh were born with LBW, representing one of the highest rates among South Asian countries. This finding underscores a significant public health challenge in Bangladesh [10].

Pregnancy‐related variables and maternal determinants have a major impact on an infant's birth weight. These factors differ greatly among various demographic, socioeconomic, and biological groups [11]. Studies have shown a correlation between LBW and factors such as, the mother's education, access to antenatal care (ANC), place of delivery, quality of health care provided during delivery, alcohol consumption, maternal anemia, malnutrition, food taboos, inadequate diet, lack of iron and folate supplementation and lack of nutritional guidance during pregnancy [4, 5, 12, 13]. Additional investigation has shown significant associations between LBW and maternal characteristics such as female sex, rising parity, young maternal age, short maternal height, low‐calorie intake, malaria, place of residence, hemoglobin level, lower socioeconomic status, and maternal smoking [6, 14, 15, 16, 17, 18]. Besides that, birth weight is found to be affected to a greater extent by gestational age [6, 16]. Following a retrospective cross‐sectional study, the likelihood of LBW significantly decreased for every increased week of gestation. Furthermore, a significant obstacle to improving maternal nutrition, which results in the delivery of LBW infants in Bangladesh, is the lack of empowerment of women [19].

Several studies have been conducted to determine the socioeconomic, demographic, and prenatal health factors that influence the prevalence of LBW in Bangladesh. However, paternal factors have been largely ignored in previous research, despite the fact that they may also play a significant role in the occurrence of LBW babies. There was a significant gap in the literature, so this study was conducted to find out how maternal and paternal factors, as well as socioeconomic, demographic, and neonatal factors, affected infants' birth weight.

## 2 Methods

### 2.1 Study Design and Population

This cross‐sectional study was conducted from July 2022 to September 2022. The study population comprised women of reproductive age (15–49 years) from eight administrative divisions of Bangladesh: Dhaka, Chittagong, Khulna, Rajshahi, Barisal, Sylhet, Mymensingh, and Rangpur. Inclusion criteria were: (a) women who had recently given birth, and (b) women who could provide either a documented or reliably recalled birth weight of their newborns.

**Figure 1:**
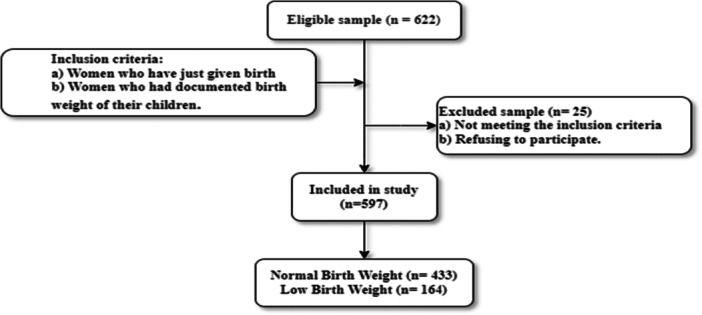
Recorded birth weight of newborns as reported by respondents.

Both institutional (hospital/clinic) and home‐based deliveries were taken into account to ensure a representative sample of childbirth settings across the country. Women who could not provide the birth weight of their newborns were excluded from the study. Figure 1 shows the participant selection flow, including eligibility, exclusions, and birth weight classification.

### 2.2 Sampling Technique and Data Collection Tool

A total of 597 samples were selected using the simple random sampling technique from a list of eligible women identified through community health worker records and local birth registries. Trained data collectors conducted structured face‐to‐face interviews using a questionnaire initially developed in English and later translated into the local language to ensure cultural and linguistic appropriateness. A pilot survey was conducted among 30 participants from the target population to assess the validity and reliability of the instrument. Based on the feedback, some items were reworded for better comprehension. These pilot participants were excluded from the final analysis to prevent potential bias.

### 2.3 Measurement Tools

Data on birth weight were gathered by asking mothers whether their child was born with a normal or low birth weight and whether the child weighed less than 2.5 kg at birth. If a birth certificate or health card were not available, mothers provided an approximate weight estimate. Birth weight is categorized as low birth weight (LBW) if < 2.5 kg and normal if ≥ 2.5 kg. All predictors were evaluated using questionnaires that were approved by the ethical committee.

### 2.4 Study Variables

The dependent variable in this study was infant birth weight, categorized as low birth weight (< 2.5 kg) and normal birth weight (≥ 2.5 kg).

The independent variables were selected after an extensive review of previous literature [5, 12, 20, 21, 22, 23, 24, 25, 26] and were grouped into three categories: neonatal, parental socioeconomic, and demographic and maternal health related variables. Characteristics related to neonatal health‐related variables included gender (male, female), birth order (first born, later born), premature birth (yes, no), and birth asphyxia (yes, no).

Parental socioeconomic variables included father's age at the time of pregnancy (< 40 years, ≥ 40 years), mother's age at the time of pregnancy (< 21 years, 21–35 years, > 35 years), socioeconomic status (low, middle, high), father's education (secondary level, higher secondary level, graduation, higher education), mother's education (secondary level, higher secondary level, graduation, higher education), father's occupation (non‐employed, employed), mother's occupation (non‐employed, employed). Demographic and maternal health‐related variables include types of family (nuclear, joint), family history of Autism disorder (no family history of ASD, family history of ASD, sibling history of ASD), father's illness (no, diabetes, other), consanguinity (not related, first degree relative), threatened abortion < 20 weeks (yes, no), specific illness during pregnancy (no, diabetes, other), maternal history of specific medicine use during pregnancy (yes, no), psychological stress of mother during pregnancy (yes, no), poor nutrition during pregnancy (yes, no), vitamin D deficiency (yes, no), mineral deficiency (yes, no), types of delivery (virginal delivery, cesarean section).

### 2.5 Statistical Analysis

The consistency and completeness of the data set were checked and then coded using Microsoft Excel. The Statistical Package for the Social Sciences (SPSS) version 26.0 was used for all statistical analyses. The characteristics of the study participants were compiled using descriptive statistics, such as frequency tables. The *χ* ^2^ test was used to investigate the associations between low birth weight and the explanatory variables. To identify significant risk factors for low birth weight, binary logistic regression was implemented as a multivariate analysis. The magnitude and precision of the associations were evaluated using the odds ratio (OR) and its 95% confidence interval (CI). The *p*‐value for statistical significance was set at 0.05, and all statistical tests were two‐sided to assess the probability of observing the results in either direction. The logistic regression model's goodness of fit was evaluated using the Hosmer–Lemeshow test.

### 2.6 Ethical Statement

This study was approved by the Noakhali Science and Technology University Ethical Committee (NSTUEC), under approval number NSTU/SCI/EC/2022/125. The study was conducted following the Declaration of Helsinki.

Written informed consent was obtained from all adult participants before data collection. For participants under the age of 18, written informed consent was obtained from a parent or legal guardian, and verbal assent was obtained from the minors themselves. As the study did not involve any medical or surgical procedure on humans, verbal consent was obtained from the mothers. A structured questionnaire was used to gather data in a community‐based setting in Bangladesh. There were no incentives offered for participation in the study; it was completely voluntary. Participants were ensured the confidentiality of their identity and given data. This study used Primary data sources, and all ethical guidelines were followed by the Demographic and Health Survey during data collection. Ethical approval was obtained from the ethical committee, and informed consent was obtained from each respondent.

## 3 Results

### 3.1 Descriptive Statistics

This study examined data from 597 mother–infant pairs in eight Bangladeshi administrative divisions. Most of the mothers were between the ages of 21 and 35 (50.4%) and had completed secondary school (34%). The majority of fathers were under the age of 40 (68.2%) and employed (99.5%), with a considerable portion (40.9%) having completed graduation‐level education. In terms of socioeconomic status, 66% of families were middle‐class, and 74% of households were nuclear families, both of which are generally in line with national trends.

First‐degree familial relationships were reported by 21.6% of the parents. The majority of the infants were firstborns (57.6%), with a slightly higher percentage of males (52.9%). 32.8% of births were premature, and 27.3% of infants experienced birth asphyxia. Cesarean section was reported by 65.2% of mothers, making it the most common delivery method. This is consistent with Bangladesh's rising national rate of C‐section deliveries.

**Figure 2:**
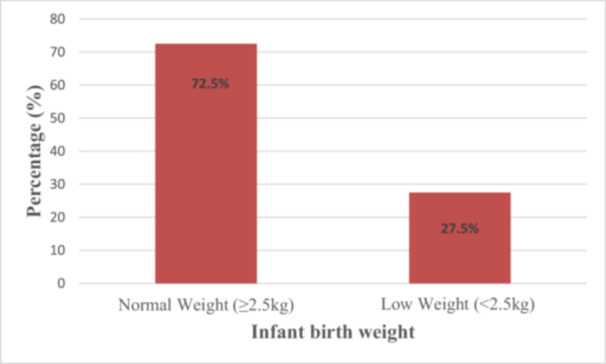
Frequency distribution of birth weight among study participants.

Mothers reported various health and nutrition‐related problems during pregnancy. Specifically, 58.6% experienced psychological stress, while 26.3%, 25.8%, and 27.3% reported nutritional, vitamin D, and mineral deficiencies, respectively. Furthermore, 32.3% of pregnant women reported having certain illnesses, and 28.8% said they were taking certain medications. 9.9% of mothers reported having previously threatened an abortion before 20 weeks of pregnancy. These physical and mental difficulties experienced by mothers could lead to complications during pregnancy and delivery. Overall, 27.5% of the infants were born with low birth weight (Figure 2).

### 3.2 Bivariate Analysis

The association between explanatory variables and low birth weight (LBW) was investigated using the *χ* ^2^ test of association (Table 1). The *χ* ^2^ test showed significant associations between LBW and premature birth, father age at the time of pregnancy, mother age at the time of pregnancy, mothers' education, maternal history of specific medicine use during pregnancy, mineral deficiencies, and father's illness.

**Table 1:** Characteristics of study participants and their relationship with birth weight.

Variable	Category	Frequency (*n*)	Percentage (%)	Infant birth weight		*χ* ^2^	*p* value	
				Low weight (*n*)	Normal weight (*n*)			
Gender	Male	316	52.9	79	237	2.05	0.152	
	Female	281	47.1	85	196			
Birth order	First born	344	57.6	89	255	1.04	0.308	
	Later born	253	42.4	75	178			
Premature birth	No	401	67.2	96	305	7.64	**0.006**	
	Yes	196	32.8	68	128			
Birth asphyxia	No	434	72.7	111	323	2.86	0.091	
	Yes	163	27.3	53	110			
Father's age at the time of pregnancy	< 40	407	68.2	99	308	6.35	**0.012**	
	≥ 40 years	190	31.8	65	125			
Mother's age at the time of pregnancy	< 21 years	86	14.4	25	61	27.29	**0.000**	
	21–35 years	301	50.4	56	245			
	> 35 years	210	35.2	83	127			
Socioeconomic status	Low	35	5.9	12	23	3.52	0.172	
	Middle	454	76	116	338			
	Higher	108	18.1	36	72			
Fathers' education	Secondary level	94	15.7	34	60	7.69	0.053	
	Higher secondary level	135	22.6	33	102			
	Graduation	244	40.9	57	187			
	Higher education	124	20.8	40	84			
Mothers' education	Secondary level	203	34	62	141	8.40	**0.038**	
	Higher secondary level	194	32.5	39	155			
	Graduation	140	23.5	42	98			
	Higher education	60	10.1	21	39			
Fathers' occupation	Not employed	3	0.5	0	3	1.14	0.285	
	Employed	594	99.5	164	430			
Mothers' occupation	Not employed	264	44.2	66	198	1.45	0.414	
	Employed	333	55.8	98	235			
Types of family	Nuclear family	442	74	118	324	0.51	0.474	
	Joint family	155	26	46	109			
Family history of autism disorder	No family history of ASD	437	73.2	124	313	1.07	0.585	
	Family history of ASD	97	16.2	26	71			
	Sibling history of ASD	63	10.6	14	49			
Consanguinity	Not related	468	78.4	135	333	2.06	0.152	
	First‐degree relative	129	21.6	29	100			
Threatened abortion < 20 weeks	No	538	90.1	149	389	0.14	0.711	
	Yes	59	9.9	15	44			
Maternal history of specific medicine use during pregnancy	No	425	71.2	130	295	7.19	**0.007**
	Yes	172	28.8	34	138		
Psychological stress of the mother during pregnancy	No	247	41.4	74	173	1.31	0.252
	Yes	350	58.6	90	260		
Poor nutrition during pregnancy	No	440	73.7	116	324	1.03	0.310
	Yes	157	26.3	48	109		
Vitamin D deficit	No	443	74.2	116	327	1.43	0.233
	Yes	154	25.8	48	106		
Mineral deficiencies	No	434	72.7	108	326	5.34	**0.021**
	Yes	163	27.3	56	107		
Types of delivery	Vaginal delivery	208	34.8	58	150	0.03	0.868
	Cesarean section	389	65.2	106	283		
Specific illness during pregnancy	No	404	67.7	115	289	1.52	0.468
	Diabetics	49	8.2	15	34		
	Others	144	24.1	34	110		
Father illness	No	423	70.9	122	301	6.96	**0.031**
	Diabetics	75	12.6	25	50		
	Others	99	16.6	17	82		

*Note:* Bold values are statistically significant.

**Figure 3:**
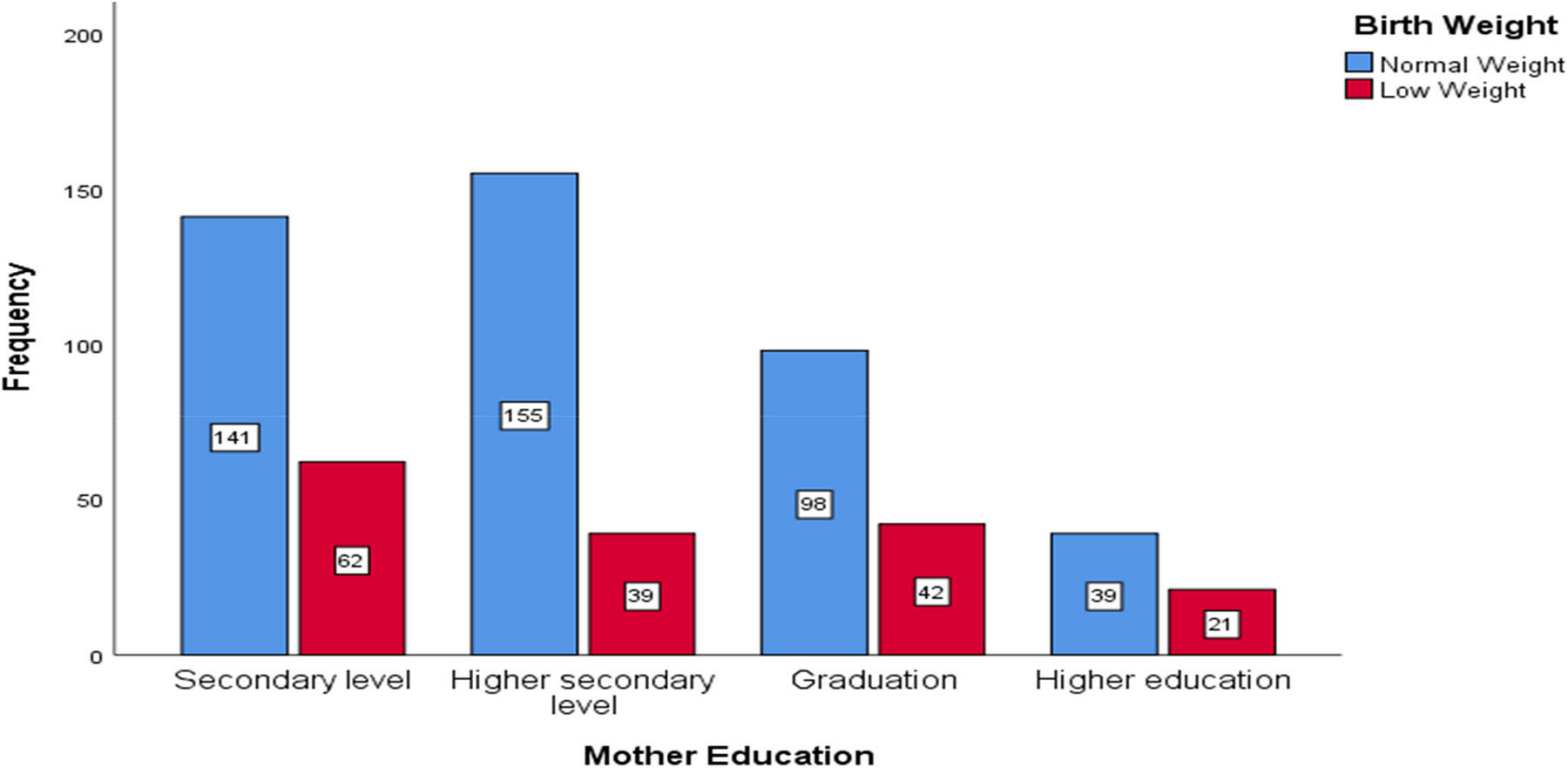
Frequency distribution of birth weight categories by maternal education level.

**Figure 4:**
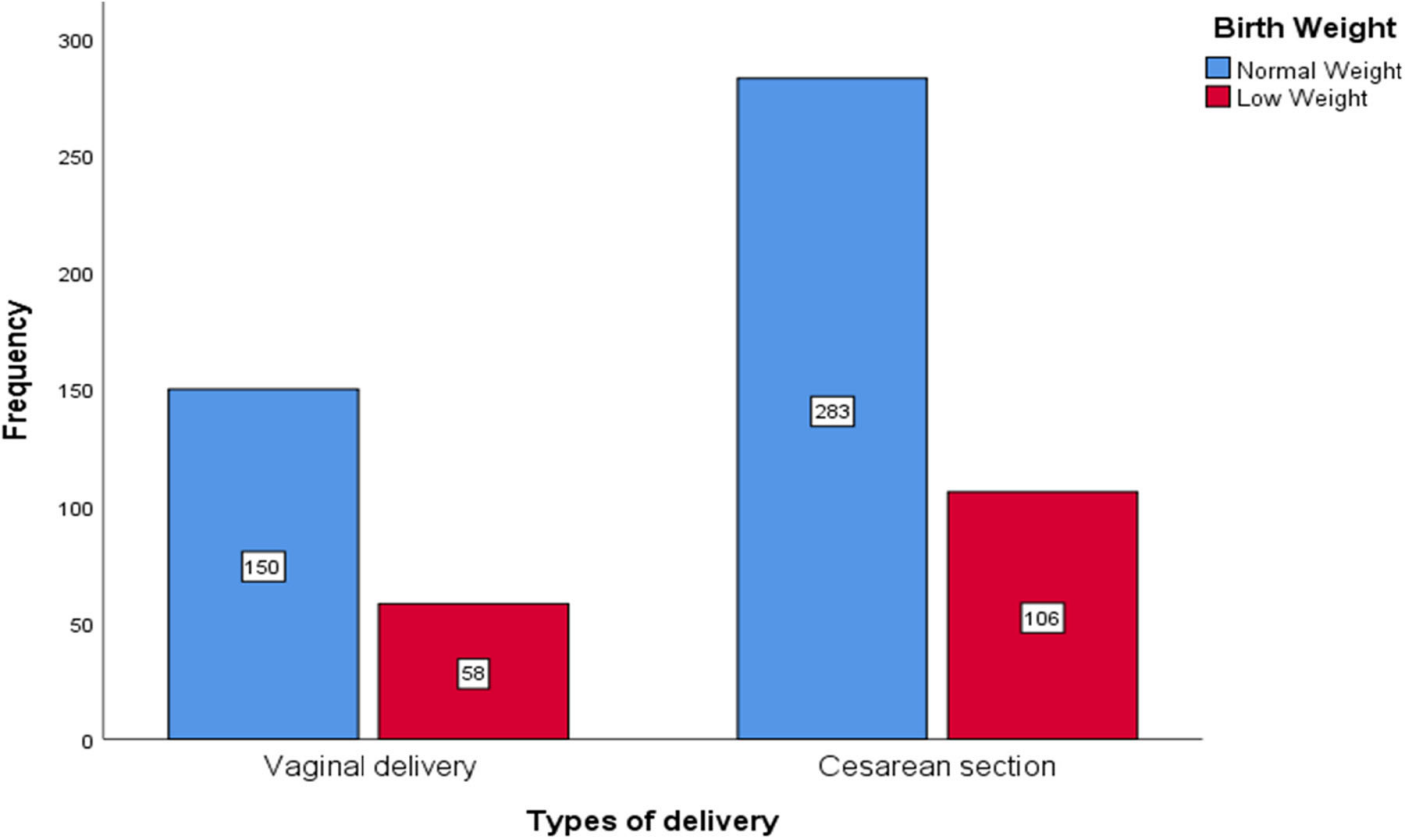
Frequency distribution of birth weight categories by types of delivery.

**Figure 5:**
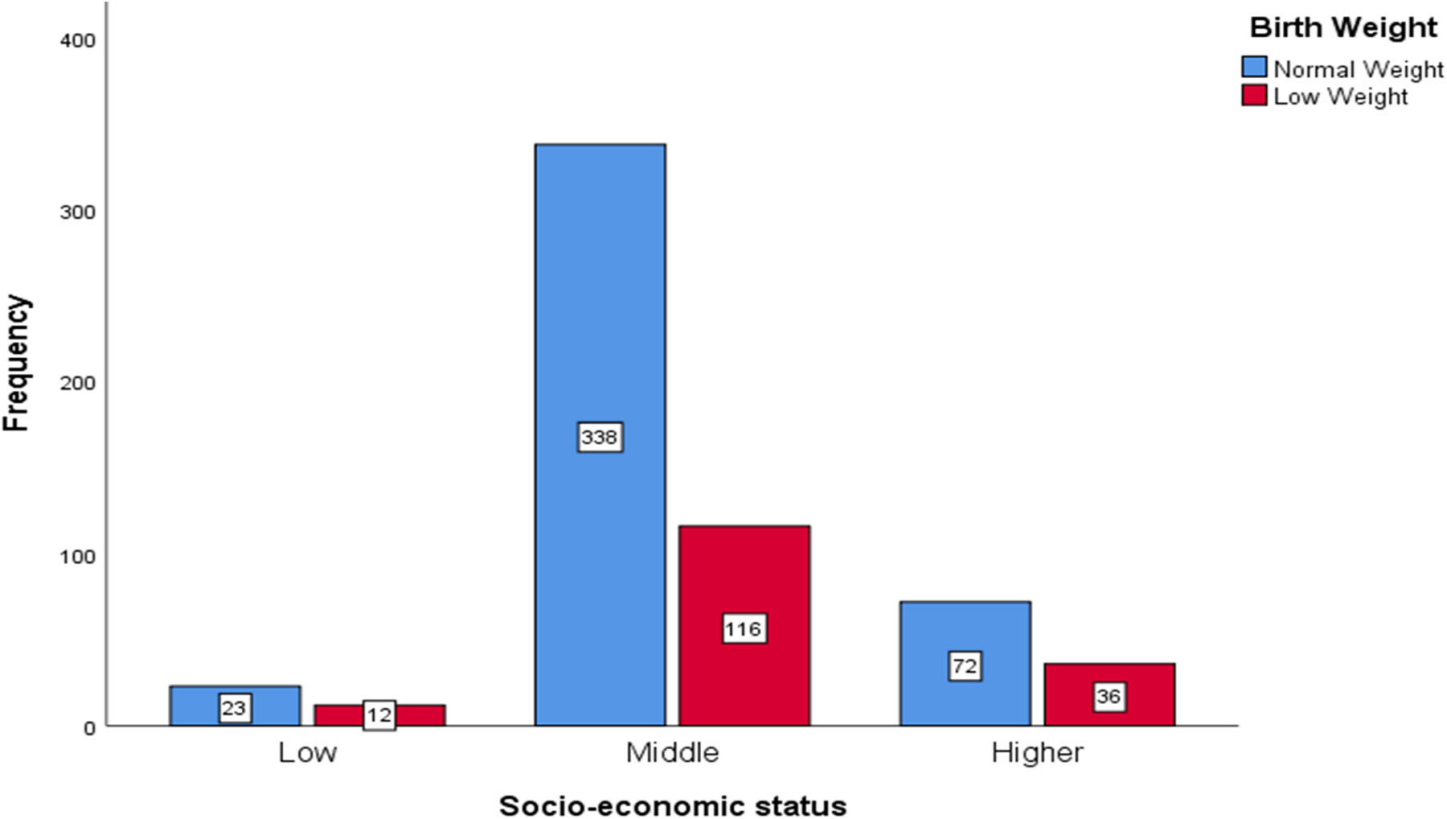
Frequency distribution of birth weight categories by socioeconomic status.

Low birth weight (LBW) and premature birth were significantly associated (*χ* ^2^ = 7.641, *p* = 0.006), suggesting that LBW is more likely to occur with premature birth. In a similar vein, there was a significant association (*χ* ^2^ = 6.354, *p* = 0.012) between the father's age at the time of pregnancy and the likelihood of having LBW children; fathers aged ≥ 40 were more likely to have such children. LBW was significantly associated with maternal age (*χ* ^2^ = 27.298, *p* < 0.001), suggesting LBW babies are less likely to be born to older mothers than to younger ones. Furthermore, LBW was significantly associated with the mother's history of using a particular medication during pregnancy (*χ* ^2^ = 7.196, *p* = 0.007), indicating that medication use during pregnancy may have an impact on the outcome of birth weight. Lastly, LBW was significantly linked to mineral deficiencies during pregnancy (*χ* ^2^ = 5.335, *p*= 0.021), highlighting the importance of maternal nutrition in determining birth weight. These results imply that the main causes of LBW are early birth, parental age, medication use, and nutritional factors like mineral deficiencies. The low birth weight (LBW) cross‐tabulations with maternal education, delivery types, and socioeconomic status are presented in Figures 3, 4, 5, respectively. Notable patterns were found despite the associations not being statistically significant. Mothers with lower levels of education were more likely to experience LBW, and a higher percentage of LBW was seen in cesarean deliveries.

### 3.3 Multivariate Analysis

Binary logistic regression analysis revealed several important risk factors for low birth weight (LBW), including premature birth, maternal age, a history of using a specific medication, and mineral deficiencies (Table 2). Compared to healthy infants, preterm infants have a twofold (OR: 2.376, 95% CI: 1.566–3.606, *p* < 0.05) increased risk of LBW. This suggests that preterm infants are 2.376 times more likely to be born with LBW than full‐term infants, and the confidence interval further supports the estimation's precision. Maternal age influences the newborn's birth weight. This finding suggests that older mothers are less likely than younger mothers to deliver babies with low birth weights. Specifically, mothers aged > 35 years have 39.6% lower odds (OR: 0.396; 95% CI: 0.252–0.624) of LBW compared with mothers under the age of 21. Older mothers are less likely to produce LBW infants when the odds ratio is less than 1. Mineral deficiency is yet another significant contributor to LBW, as minerals are necessary for an infant's healthy development in the womb. LBW babies are 1.838 times more likely to be born to women who lack certain minerals (OR: 1.838, 95% CI: 1.200–2.816, *p* = 0.005). It means that mineral deficiencies elevate the likelihood of LBW, and the confidence interval suggests that this risk is precisely estimated. Moreover, women with a history of using specific medications during pregnancy have 39.1% lower odds of LBW in comparison to those without such a history (OR: 0.609; 95% CI: 0.373–0.995; *p* < 0.05). This implies that specific medications may have a protective effect on birth weight.

**Table 2:** Binary logistic regression analysis of selected variables on newborn birth weight.

			95% Confidence interval		
Variables	Categories	Odds ratio	Lower	Upper	*p* value
Premature birth	No (Reference)	1	—	—	—
	Yes	2.38	1.57	3.61	0.000
Father's age at the time of pregnancy	< 40 years (reference)	1	—	—	—
	≥ 40 years	1.45	0.95	2.21	0.086
Mother's age at the time of pregnancy	< 21 years (reference)	1	—	—	—
	21–35 years	0.66	0.36	1.19	0.163
	> 35 years	0.39	0.25	0.62	0.000
Mothers' education	Secondary level (reference)	1	—	—	—
	Higher secondary level	0.75	0.39	1.45	0.398
	Graduation	0.52	0.26	1.03	0.060
	Higher education	0.94	0.48	1.87	0.867
Fathers' illness	No (reference)	1	—	—	—
	Diabetics	1.40	0.75	2.61	0.285
	Others	1.51	0.70	3.24	0.288
Maternal history of specific medicine use during pregnancy	No (reference)	1	—	—	—
	Yes	0.60	0.37	0.99	0.048
Mineral deficiencies	No (reference)	1	—	—	—
	Yes	1.84	1.20	2.82	0.005
Hosmer and Lemeshow Test = 5.96		*p* = 0.652			

*Note:* The results of the Hosmer–Lemeshow test showed a good model fit, with *χ* ^2^ = 5.960 and *p* value = 0.652. This shows that the logistic regression model captures the observed data in a sufficient way.

## 4 Discussion

Birth weight is a critical determinant of a newborn's survival, growth, and long‐term health. In Bangladesh, low birth weight (LBW) remains a significant public health concern. This study aimed to identify parental socioeconomic, demographic, and health‐related factors associated with LBW among newborns. The cross‐sectional study analyzed data from 597 newborns, revealing a 27.5% prevalence of LBW. This rate is higher than the previously reported incidence of 10.6% [18] LBW babies. Earlier, the frequency of LBW was reported to range between 15% and 30% in South Asian countries [18]. In addition, a study conducted in Bangladesh found that 20% of newborns had low birth weights [23], while another study reported a prevalence of 14.5% [27]. The increase in LBW prevalence in this study may be attributed to factors such as premature birth, younger maternal age, the absence of maternal medication use during pregnancy, and maternal mineral deficiencies.

The result of this study showed that women who gave birth prematurely had a significantly higher risk of having LBW babies than women who had term deliveries. This finding is consistent with studies from Nepal [21, 28, 29], Ethiopia [30], Kenya [31], and India [32], which identified premature birth as a significant predictor of LBW. Similarly, rural India studies have reported that 6.4% of LBW babies were preterm [33].

An additional significant factor was the mother's age. Younger mothers had a higher chance of having LBW babies than older mothers, possibly due to their limited experience and increased vulnerability to pregnancy complications. This is consistent with previous findings showing that younger mothers were significantly more likely to deliver LBW babies than older mothers [22, 23, 24]. However, conflicting results exist. For example, studies from Afghanistan [20] and Nepal [21, 29] found no significant association between maternal age and LBW. Additionally, another study revealed that advanced maternal age at childbirth had a noticeably higher risk of LBW (35–49 years) [34]. These disparities could be caused by variations in the quality and accessibility of maternal healthcare services, lifestyles, and dietary habits [35, 36]. For example, adequate antenatal care reduces the age‐related risk of low birth weight by ensuring proper monitoring, nutrition, and timely interventions, whereas limited access may result in poorer outcomes [37].

Another significant factor identified was maternal medication use during pregnancy. In our study, low birth weight (LBW) babies were less likely to be born to women who reported taking certain medications, such as antacids, antibiotics, and antifungal drugs. These medications are frequently prescribed to prevent or manage pregnancy‐related complications, which may indirectly contribute to improved birth outcomes [38]. However, this relationship should be interpreted with caution. Pregnancy medication use is a potential confounding factor because it can serve as a stand‐in for antenatal care quality, access to healthcare, or underlying maternal health conditions. In fact, some research has shown conflicting findings; for instance, prenatal antibiotic exposure has been associated with reduced birth weight in certain populations [39], and certain topical antiglaucoma medications (excluding beta‐blockers) have been associated with an increased risk of LBW [40].

Our research found an association between mineral deficiency and low birth weight (LBW) in newborns, implying that babies with LBW are more likely to be born to mineral‐deficient mothers during pregnancy. An earlier study found that nutrient‐poor gestational diets can cause maternal and fetal malnutrition, with symptoms apparent from birth through infancy. These deficiencies are associated with small‐for‐gestational‐age (SGA) births, LBW infants, and premature births (gestational age < 37 weeks) [41]. Iron and folic acid deficiencies are well known to be important contributors to fetal growth restriction and unfavorable birth outcomes [42, 43]. Another study also reported that mothers who did not take folic acid and iron supplementation during pregnancy were more likely to give birth to LBW neonates than those who took supplementation [25]. Folic acid insufficiency is linked to poor fetal development and an elevated risk of preterm birth and LBW, whereas iron deficiency anemia can hinder oxygen transport to the fetus, resulting in intrauterine growth restriction [44, 45]. When considered collectively, these studies offer strong evidence suggesting that mineral deficiency can significantly impact newborn health. It is crucial to guarantee sufficient maternal nutrition through dietary intake and supplementation in light of these associations. Maternal health awareness campaigns, nutritional counseling, and prenatal supplementation programs are examples of public health initiatives that may help lower the prevalence of LBW. Pregnant women in low‐income countries frequently consume diets deficient in essential nutrients due to financial constraints, which increases the risk of LBW in infants [46]. To address these issues, it should be a top priority to reinforce community‐based maternal health services, increase access to prenatal supplements, and integrate targeted nutritional programs.

This study found that the rate of cesarean deliveries among LBW infants was higher, though it was not statistically significant. Similar patterns were noted in Brazil, where LBW was more common in caesarean deliveries, particularly among women with higher education. This finding may be a reflection of social and healthcare disparities [47]. Another study found that cesarean delivery is associated with LBW, possibly as a result of illnesses or issues that caused an early delivery [48].

There are various limitations to this study. Despite adjusting for confounders, the inclusion of adolescent mothers may introduce bias because they differ from adult mothers in terms of socioeconomic characteristics and health behaviors. Recall bias may affect self‐reported data, particularly when it comes to recording birth weight and maternal health history. Despite efforts to standardize procedures, interobserver variability in data collection may have affected the accuracy of the results. Furthermore, frequency weights were not applied to the *χ* ^2^ tests in this study, which might have limited how broadly the results can be applied. The robustness of the logistic regression results could be impacted by the absence of a multicollinearity assessment among the independent variables. In addition, this study can only establish associations rather than causal relationships because of its cross‐sectional design. To better understand these relationships and guide successful public health initiatives, more longitudinal or interventional research is required. Regardless of these drawbacks, the study provides insightful information about the variables affecting LBW in Bangladesh.

## 5 Conclusion

Despite Bangladesh being a lower‐middle‐income nation with significantly reduced mother and infant mortality, the burden of low birth weight (LBW) is still relatively high. It is a significant contributor to infant mortality. Achieving Bangladesh's sustainable development goal relating to neonatal healthcare may be significantly hampered by this situation. This study is concerned with helping to avoid adverse maternal circumstances and infants' LBW in Bangladesh. The findings of this study revealed that premature birth, maternal age, maternal history of specific medication during pregnancy, and maternal mineral deficiency significantly impact the low birth weight of newborn babies. An elevated risk of LBW has been associated with deficiencies in vital nutrients, including vitamins, folic acid, and iron. Therefore, improving birth outcomes requires making sure that mothers receive enough nutrition through appropriate dietary interventions and supplements. Preventing premature deliveries is one of the best ways to avoid LBW infants. It can be suggested that the best prevention of premature birth leading to LBW among newborns is regular medical supervision during pregnancy. Proper care and concern should be given to all pregnancies. Based on the study's findings, we recommend that volunteers, family members, healthcare professionals, and policymakers focus on nutritional values, especially before and during pregnancy.

## Author Contributions

**Md Rasel Hossain:** conceptualization, investigation, funding acquisition, writing – original draft, writing – review and editing, visualization, validation, methodology, formal analysis, project administration. **Susmita Begum:** funding acquisition, writing – original draft, investigation, visualization, validation, writing – review and editing, project administration, formal analysis, resources. **Mahmuda al Neyma:** investigation, writing – review and editing, writing – original draft, visualization, validation, methodology, software, formal analysis, project administration. **Kabir Hossain:** writing – original draft, writing – review and editing, investigation. **Mohammad Omar Faruk:** conceptualization, investigation, funding acquisition, writing – original draft, writing – review and editing, visualization, methodology, software, formal analysis, and supervision. **Sorif Hossain:** conceptualization, investigation, funding acquisition, writing – original draft, writing – review and editing, visualization, validation, formal analysis, project administration, resources, supervision. **Humayra Afnan:** investigation, funding acquisition, writing – original draft, writing – review and editing, visualization, validation, formal analysis, project administration, resources.

## Ethics Statement

This study was approved by the Noakhali Science and Technology University Ethical Committee (NSTUEC), under approval number NSTU/SCI/EC/2022/125. The study was conducted following the Declaration of Helsinki. Written informed consent was obtained from all adult participants before data collection. For participants under the age of 18, written informed consent was obtained from a parent or legal guardian, and verbal assent was obtained from the minors themselves. As the study did not involve any medical or surgical procedure on humans, verbal consent was obtained from the mothers. A structured questionnaire was used to gather data in a community‐based setting in Bangladesh. There were no incentives offered for participation in the study; it was completely voluntary. Participants were ensured the confidentiality of their identity and given data. This study used Primary data sources, and all ethical guidelines were followed by the Demographic and Health Survey during data collection. Ethical approval was obtained from the ethical committee, and informed consent was obtained from each respondent.

## Consent

All respondents gave their consent before collecting the data, which was confirmed by the enumerators. All respondents provided consent for the publication of the survey results. All authors have read the manuscript and provided their consent to publish this article.

## Conflicts of Interest

The authors declare no conflicts of interest.

## Transparency Statement

The lead authors, Md. Rasel Hossain and Sorif Hossain, affirm that this manuscript is an honest, accurate, and transparent account of the study being reported; that no important aspects of the study have been omitted; and that any discrepancies from the study as planned (and, if relevant, registered) have been explained.

## Data Availability

The data that support the findings of this study are available from the corresponding author upon reasonable request. The code for this study will be provided on request.
